# Role of Adaptor Proteins in Secretory Granule Biogenesis and Maturation

**DOI:** 10.3389/fendo.2013.00101

**Published:** 2013-08-14

**Authors:** Mathilde L. Bonnemaison, Betty A. Eipper, Richard E. Mains

**Affiliations:** ^1^Department of Molecular, Microbial and Structural Biology, University of Connecticut Health Center, Farmington, CT, USA; ^2^Department of Neuroscience, University of Connecticut Health Center, Farmington, CT, USA

**Keywords:** regulated secretory pathway, maturation, cargo, AP-1, GGA, PACS-1, prohormone

## Abstract

In the regulated secretory pathway, secretory granules (SGs) store peptide hormones that are released on demand. SGs are formed at the *trans*-Golgi network and must undergo a maturation process to become responsive to secretagogues. The production of mature SGs requires concentrating newly synthesized soluble content proteins in granules whose membranes contain the appropriate integral membrane proteins. The mechanisms underlying the sorting of soluble and integral membrane proteins destined for SGs from other proteins are not yet well understood. For soluble proteins, luminal pH and divalent metals can affect aggregation and interaction with surrounding membranes. The trafficking of granule membrane proteins can be controlled by both luminal and cytosolic factors. Cytosolic adaptor proteins (APs), which recognize the cytosolic domains of proteins that span the SG membrane, have been shown to play essential roles in the assembly of functional SGs. Adaptor protein 1A (AP-1A) is known to interact with specific motifs in its cargo proteins and with the clathrin heavy chain, contributing to the formation of a clathrin coat. AP-1A is present in patches on immature SG membranes, where it removes cargo and facilitates SG maturation. AP-1A recruitment to membranes can be modulated by Phosphofurin Acidic Cluster Sorting protein 1 (PACS-1), a cytosolic protein which interacts with both AP-1A and cargo that has been phosphorylated by casein kinase II. A cargo/PACS-1/AP-1A complex is necessary to drive the appropriate transport of several cargo proteins within the regulated secretory pathway. The Golgi-localized, γ-ear containing, ADP-ribosylation factor binding (GGA) family of APs serve a similar role. We review the functions of AP-1A, PACS-1, and GGAs in facilitating the retrieval of proteins from immature SGs and review examples of cargo proteins whose trafficking within the regulated secretory pathway is governed by APs.

## The Regulated Secretory Pathway

Neuroendocrine cells synthesize, process, and store peptide hormones so that they are available for secretion upon demand (1). These professional secretory cells devote as much as half of their total protein synthesis to the production of a single hormone (2). The regulated secretory pathway allows intracellular storage of peptide hormones until an external stimulus triggers exocytosis of the secretory granules (SGs) that contain the peptides. Neuroendocrine tumors and metabolic disease are linked to defects in hormone secretion, observed via an increase in circulating hormone levels due to impaired intracellular storage or cellular response. The alterations which result in loss of storage and secretagogue responsiveness are poorly understood.

**Figure 1:**
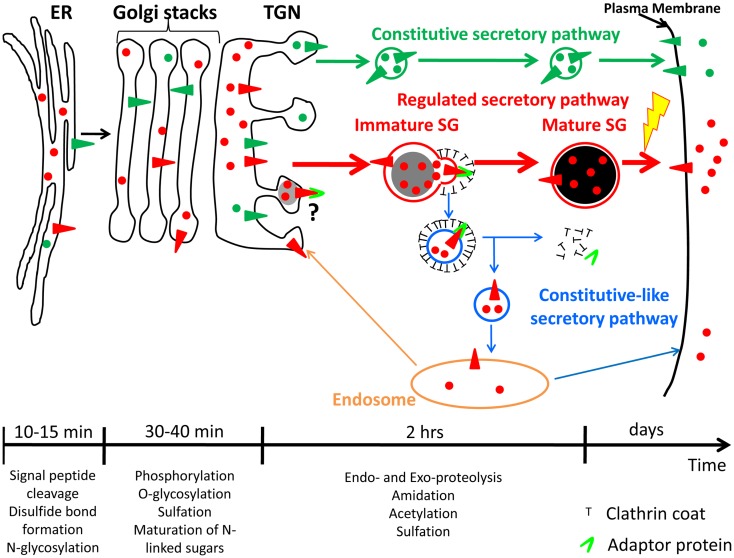
**Constitutive and regulated secretory pathways**. Soluble and membrane proteins are represented with circles and triangles, respectively. Constitutive secretory pathway proteins are green; regulated secretory pathway proteins are red. Examples of post-translational modifications that occur in each organelle are shown. The time-scale is representative of the time spent by hormones and hormone processing enzymes in each organelle of the regulated secretory pathway. Until newly synthesized proteins reach the *trans-*Golgi network (TGN), proteins from both secretory pathways share the same compartments. Secretory proteins are sorted into constitutive secretory vesicles or immature secretory granule (SGs) in the TGN. Immature SGs undergo a maturation process to become mature SGs. The yellow lightning bolt represents the external stimulus triggering secretion of mature SG content. The maturation process involves remodeling of immature SG membranes, generating the constitutive-like secretory pathway (blue); the fate of the vesicles leaving immature SGs is not clear; return to an endosomal compartment from which proteins can be secreted in a constitutive-like fashion or recycled to the TGN is shown. ER, endoplasmic reticulum.

Peptide hormones are first synthesized as inactive precursors. The signal peptide found at the N-terminus of the preprohormone is recognized by signal recognition particle, which stops translation and directs entry of the nascent preprohormone into the lumen of the endoplasmic reticulum (3); removal of the signal peptide by signal peptidase yields the prohormone. Most proteins which are going to be secreted undergo this step. The endoplasmic reticulum is also the site at which disulfide-bond formation and N-linked glycosylation occur (Figure 1) (3, 4, 5). Professional secretory cells have developed specialized sensing mechanisms to avoid triggering the endoplasmic reticulum stress pathway, which can lead to cell death; for example, increased expression of Stress-associated Endoplasmic Reticulum Protein 1 (SERP1) prevents endoplasmic reticulum stress in the anterior pituitary and pancreas (6).

After exiting the endoplasmic reticulum, prohormones are transported to the Golgi apparatus, where additional post-translational modifications such as oligosaccharide maturation and phosphorylation can occur (Figure 1) (5, 7). When they reach the *trans*-Golgi network (TGN), prohormones, and their processing enzymes are concentrated into granules budding from the TGN; these structures presumably represent newly forming SGs (1). These new SGs are immature and must undergo a maturation process before they are capable of secreting peptide hormone in response to secretagogue. Maturation involves remodeling of the immature SG membrane by removal of non-regulated secretory proteins and excess membrane; this process involves clathrin-coated vesicles mediated by adaptor proteins (APs) (Figure 1) (8, 9), acidification of the lumen, aggregation of content proteins and, at least in some cell systems, fusion of immature SGs (10, 11). The final post-translational modifications needed to generate bioactive peptide hormones occur in immature and mature SGs (12, 13). Mature SGs, which appear dense in the electron microscope and during sucrose density gradient centrifugation, can contain more than 300 mg/ml protein, largely peptide hormones (14). Unlike constitutively secreted proteins, which are found in the extracellular compartment within minutes after exit from the TGN, it takes about 90 min for peptide hormones to go from the TGN to mature SGs (Figure 1) (15, 16). In addition, mature SG content can be stored for many days before being secreted into the extracellular compartment in response to a stimulus (Figure 1) (17, 18); proteins and peptides stored in the regulated secretory pathway are released at a low rate (basal secretion) even in the absence of secretagogue (19, 20).

The study of immature SGs remains a challenge due to their transient role as intermediates between the TGN and mature SGs. Morphologists describe immature SGs as vacuoles found in close proximity to the TGN which contain dense material surrounded by a loose membrane with a partial clathrin coat, while biochemists distinguish immature SGs from mature SGs by their inability to respond to secretagogue or by their release of incompletely processed newly synthesized products (14, 21). Early studies using ^35^SO_4_ to label sugars and Tyr residues demonstrated high K^+^/Ca^2+^-stimulated release of ^35^SO_4_-labeled SG components as soon as 15 min after synthesis (16); it is not clear how to relate ^35^SO_4_ labeling to biosynthetic or endocytic trafficking. In the end, what controls and triggers the formation and maturation of immature SGs remains unclear. Both soluble and membrane proteins destined for the regulated secretory pathway must enter immature SGs when they exit the TGN, but how the trafficking of soluble and membrane proteins is coordinated is still under debate.

## Formation of Immature SGs

Proteins destined for the regulated secretory pathway are sorted in the TGN and in immature SGs. Although the sorting mechanisms are not completely understood, the diverse biophysical and biochemical properties of soluble and membrane proteins suggest that they are targeted to the regulated secretory pathway through different mechanisms. The TGN is a cellular crossroad; departing proteins can enter vesicles targeted to endosomes, lysosomes, endoplasmic reticulum, or the plasma membrane (1, 22). One of the first studies demonstrating sorting of regulated secretory proteins at the TGN was performed using a cell-free system from PC12 cells, a neuroendocrine tumor cell line: vesicles budding from the TGN contained either heparin sulfate proteoglycan, a soluble protein of the constitutive secretory pathway, or secretogranin II, a soluble protein of the regulated secretory pathway (23).

### View from the luminal side

**Figure 2:**
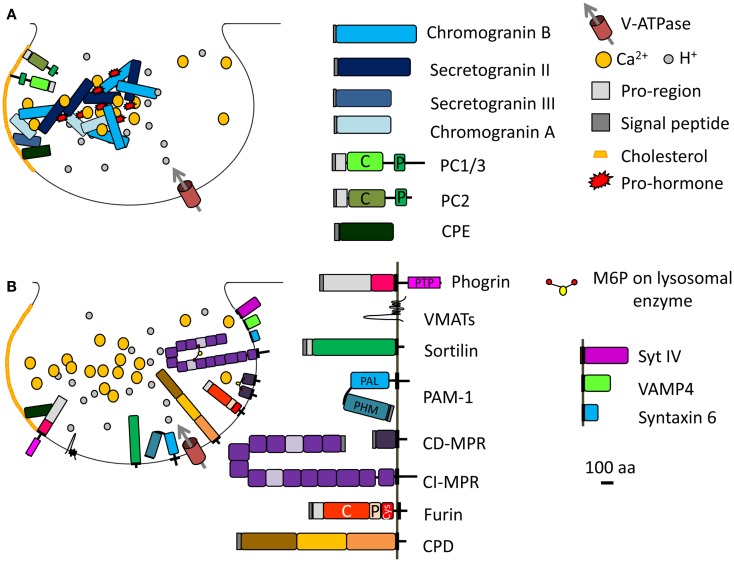
**Immature SG formation at the TGN**. **(A)** Granins and prohormones aggregate in response to the high calcium, mildly acidic conditions in the TGN. Several soluble proteins interact with cholesterol or cholesterol-rich membranes; their sorting into immature SGs depends on this interaction. **(B)** Membrane proteins identified in immature SGs and discussed in the main text are shown; their cytosolic tails can interact with adaptor proteins. The SNARE and synaptotagmin proteins that enter immature SGs (synaptotagmin IV, VAMP4, syntaxin 6) are replaced during the maturation process. Proteins known to enter immature SGs are drawn approximately to scale (except for the vacuolar proton pump, V-ATPase, and VMATs). PC, prohormone convertase; C, catalytic domain; P, P domain; PTP, protein-tyrosine phosphatase; PHM, peptidylglycine α-hydroxylating monooxygenase; PAL, peptidylglycine α-amidating lyase; CI-MPR contains 15 domains; CPD contains three similar domains; Cys, cysteine-rich domain.

The mildly acidic pH (pH 6.4) and high calcium (1–10 mM) environment of the TGN can induce aggregation of selected proteins (e.g., secretogranin II, chromogranin B, and prolactin) destined for the regulated secretory pathway, resulting in their segregation from the constitutive secretory pathway (Figure 2A) (24–26). Other regulated SG proteins bind specific lipids in the TGN, resulting in their sorting and entry into immature SGs; prohormone convertase 1/3 and prohormone convertase 2 interact with lipid rafts and secretogranin III binds to cholesterol (Figure 2A) (27–29). Indeed, chromogranin A, which enhances prohormone aggregation, interacts at the TGN with secretogranin III, and thus with cholesterol-rich membranes (Figure 2A) (28, 30). If the interaction of chromogranin A with secretogranin III is blocked, chromogranin A is not sorted correctly (31). Finally, a role for receptor-mediated sorting of regulated secretory proteins exiting the TGN has been considered. Carboxypeptidase E was proposed as a prohormone sorting receptor because it interacts with the N-terminal region of proopiomelanocortin (POMC), which was previously reported to serve as a sorting domain (15, 32). This conclusion is controversial because the sorting of proinsulin, luteinizing hormone, and follicle stimulating hormone does not depend on carboxypeptidase E (33, 34). Although the sorting of cargo upon binding to a receptor is an attractive concept, SG protein sorting appears to involve multiple processes. Carboxypeptidase E was recently shown to interact with phogrin, a SG membrane protein of the Insulinoma Associated protein 2 (IA-2) family; this interaction involves the pro-region of the luminal domain of phogrin and mature carboxypeptidase E (Figure 2B). When one binding partner is missing, the other does not accumulate in SGs, instead localizes to the perinuclear region; the sorting of carboxypeptidase E and phogrin at the TGN is inter-dependent (35).

### View from the cytosolic side

#### Sorting signals contributed by SG membrane protein trafficking

Membrane proteins cannot aggregate as extensively as soluble proteins. The identification of trafficking signals in the cytosolic domains of endocytic cargo led to the postulate that the cytosolic domains of SG membrane proteins would carry signals to ensure their entry into immature SGs (Figure 2B). Indeed, deletion or mutation of the cytosolic domain of phogrin results in a decrease in its entry into SGs (36, 37). Similar observations were made for peptidylglycine α-amidating monooxygenase 1 (PAM-1) (Figure 2B). PAM-1 is a bifunctional enzyme catalyzing the amidation of glycine-extended peptides, rendering them bioactive. Exogenous expression of a truncated PAM-1 protein lacking its cytosolic domain resulted in its inefficient storage in SGs. Metabolic labeling revealed that 20–40% of the newly synthesized truncated PAM protein entered the regulated secretory pathway, but endocytic trafficking and SG re-entry of the truncated PAM-1 protein were eliminated (38). The cytosolic domain of PAM-1, which is highly phosphorylated, interacts with several cytosolic proteins (39). Two sites in the cytosolic domain of PAM-1 are phosphorylated by casein kinase II (CKII) (Table 1); both sites were mutated to Asp (PAM-1/TS/DD), to try to mimic phosphorylation, or to Ala (PAM-1/TS/AA) to prevent phosphorylation. When expressed in AtT-20 corticotrope tumor cells, exogenous PAM-1/TS/DD entered immature SGs more efficiently than PAM-1, while PAM-1/TS/AA did not enter immature SGs efficiently and was degraded (40). This study suggests that phosphorylation of the cytosolic tail of PAM-1 enhances its entry into immature SGs.

**Table 1:** **Summary of known motifs for membrane proteins in SGs**.

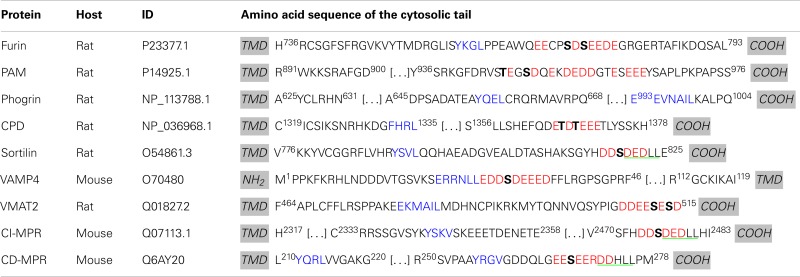

*Protein, host, and ID are shown. Acidic clusters are in red. CKII sites are in bold. AP-1 and AP-1-like sites are in blue and GGA sites are underlined in green. TMD, transmembrane domain; COOH, C-terminus of the protein; NH_2_, N-terminus of the protein. The gray shade and italic font was to help the reader distinguish COOH or TMD or NH_2_ from the amino acid sequence*.

Similar conclusions were reached in studies of two different neurotransmitter transporters, Vesicular Monoamine Transporter 2 (VMAT2) and Vesicular Acetylcholine Transporter (VAChT). Using the pH gradient established by the vacuolar proton pump, VMAT2, and VAChT translocate monoamines and acetylcholine, respectively, from the cytosol into the lumen. VMAT2 and VAChT enter two different types of vesicles in PC12 cells: VMAT2 is preferentially targeted to SGs while VAChT is found in synaptic-like microvesicles (Figure 2B) (41). The cytoplasmic domain of the VAChT contains a di-leucine motif with adjacent conserved Glu and Ser residues. Phosphorylation of this Ser by protein kinase C or mutation to a phosphomimetic residue results in a preference for VAChT entry into SGs, rather than into synaptic-like microvesicles (41). Additionally, VMAT2 contains two conserved Glu residues upstream of its di-leucine-like motif (Table 1, in blue); mutation of these Glu residues into Ala results in accumulation of VMAT2 in synaptic-like microvesicles (41).

These studies suggest that the sorting of membrane proteins into the regulated secretory pathway requires cytosolic signals and/or luminal/transmembrane signals. The negatively charged region within the cytosolic domain of membrane proteins is probably required for efficient sorting and entry into SGs. Since the cytosolic domain is involved in their sorting, cytosolic proteins must come into play in the formation of immature SGs and protein sorting in the regulated secretory pathway.

#### ADP-ribosylation factor 1 is required for SG biogenesis

**Figure 3:**
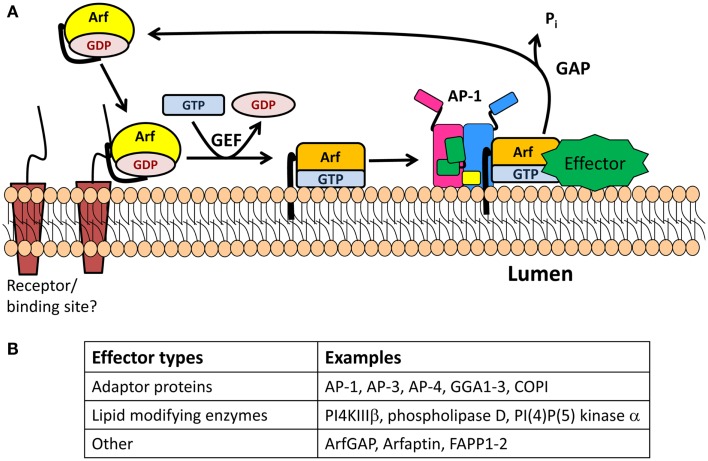
**The Arf/GEF/GAP cycle**. **(A)** GDP-Arf is cytosolic and is thought to be recruited to its target membrane by interaction with a receptor. The exchange of Arf-bound GDP for GTP is catalyzed by an Arf-GEF, which induces a conformational change in Arf and release of GDP. GTP-bound Arf binds to the membrane through its N-terminal myristoylated amphipathic helix and recruits cytosolic proteins which function in membrane trafficking (effectors). One of these effectors is an Arf-GAP, which promotes the GTPase activity of Arf, resulting in its detachment from the membrane. **(B)** Table identifying type of effectors.

In the 1990s, the cytosolic protein ADP-ribosylation factor 1 (Arf1), a member of the ADP-ribosylation family (Arf), was shown to promote the formation of immature SGs using cell-free systems from PC12 and GH_3_ cells, two neuroendocrine cell lines (42, 43). Arf proteins, which belong to the Ras superfamily of small GTPases, were originally identified as necessary for the ADP-ribosylation reaction catalyzed by cholera toxin (44). Despite their names, the cellular function of Arfs does not involve ADP ribosylation, but rather membrane trafficking (45). Arf proteins are divided into three classes: class I (Arf1, Arf2, and Arf3), class II (Arf4 and Arf5), and class III (Arf6). Class I and class II Arfs are present at the Golgi and their main function is to regulate Golgi trafficking, while Arf6 functions at the plasma membrane and in the endocytic pathway. Arfs exist in two states: GDP-bound Arf is cytosolic and inactive while GTP-bound Arf is membrane-associated and active (Figure 3) (45, 46). All Arfs contain an N-terminal myristoylated amphipathic helix, which allows their association with membranes. This region is buried in a hydrophobic pocket when GDP is bound, but becomes accessible to membranes when GTP replaces GDP, triggering conformational changes in the switch 1 and switch 2 regions which surround the nucleotide binding site (45–47). A resident TGN protein, cargo, or lipid may interact with GDP-bound Arf1, bringing it near the TGN (Figure 3). In the context of COPI vesicles, which are involved in retrograde transport from the Golgi to the endoplasmic reticulum, p23, a transmembrane resident Golgi protein, is thought to recruit GDP-bound Arf (48).

The removal of GDP and binding of GTP is catalyzed by a guanine nucleotide exchange factor (GEF) (Figure 3) (45, 46). The two Arf GEFs identified at the TGN are BIG1 and BIG2 (brefeldin A inhibited GEFs). Interestingly, GBF1, a GEF known to be involved in the GDP/GTP exchange of Arf4 and Arf5 in the *cis*-Golgi compartment, has recently been identified on the TGN membrane (49). BIG1 and BIG2 are recruited to the TGN membrane upon binding to GTP-bound Arf4 and Arf5, whose nucleotide exchange was mediated by GBF1 (49). Both BIGs contain a Sec7 domain, which is the active site for the nucleotide switch and the target of brefeldin A, a fungal product. Treatment of neuroendocrine and exocrine cells with brefeldin A blocks the formation and maturation of immature SGs but does not alter mature SG exocytosis (50, 51).

Once nucleotide exchange has occurred, GTP-bound Arf recruits APs, enzyme modifying lipids, and effectors to the membrane before reacting with a GTPase activating protein (GAP) that promotes hydrolysis of GTP to GDP and release of Arf from the membrane (Figure 3). The Arf-GAP family is composed of 24 members, each with a GAP domain essential for its activity on Arf (45). GAP activity is modulated by the presence of coat proteins previously recruited by the GTP-bound Arf. When COPI is bound to Arf1, GAP activity is increased (52). Golgi-localized, γ-adaptin ear containing, Arf-binding (GGA) 3 is a coat protein involved in TGN-to-endosome transport. In contrast, when Arf1 recruits GGA3, it blocks the hydrolysis of GTP-bound to Arf1 (53).

**Figure 4:**
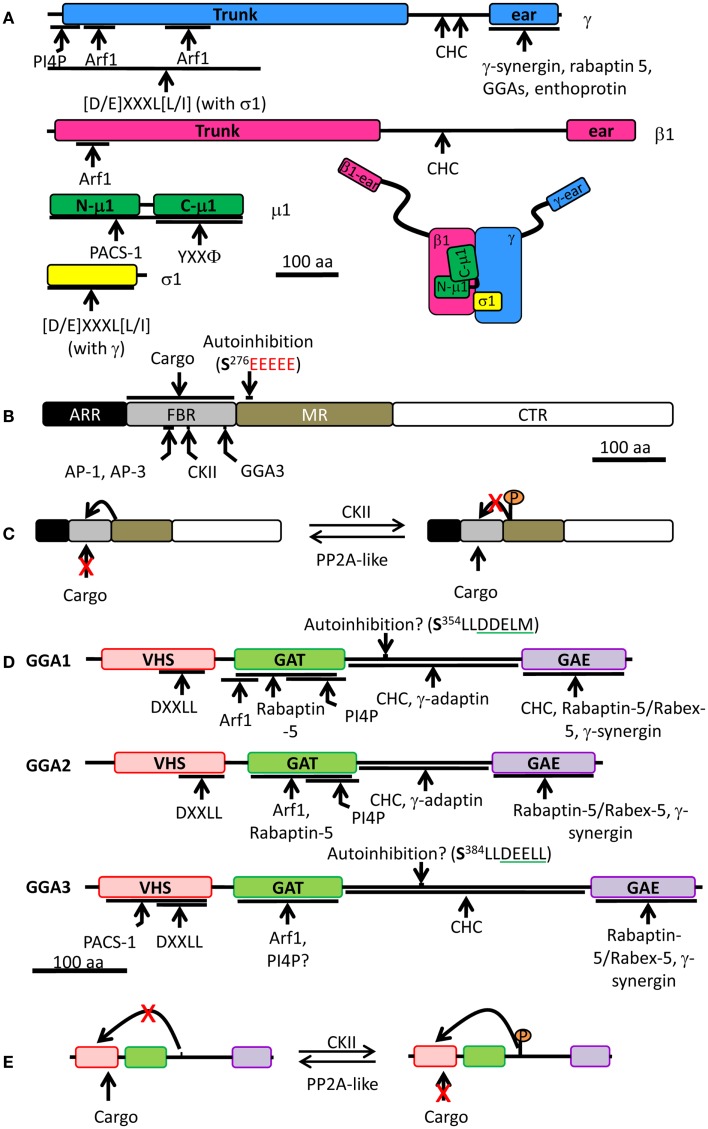
**Schematic of the AP-1 complex, PACS-1, and mammalian GGAs**. **(A)** AP-1 is made of four different subunits: γ (blue), β1 (pink), μ1 (green), and σ1 (yellow). For each subunit, binding partners or binding motifs that are recognized are indicated; Φ indicates a hydrophobic residue; X can be any amino acid. The overall organization of the complex is shown at the bottom right. **(B)** PACS-1 is composed of four domains: ARR (atrophin-1-related region, black), FBR (furin binding region, gray), MR (middle region, tan), and CTR (C-terminus region, white). The autoinhibition sequence in the MR is shown (CKII phosphorylation site is in bold and the acidic cluster in red), along with the CKII binding site in the FBR. AP-1 and AP-3 interact with the same site in the FBR while GGA3 interacts with a different site. The FBR is also responsible for interactions with cargo. **(C)** Autoinhibition mechanism: the autoinhibition domain binds FBR, preventing PACS-1 interaction with cargo. When CKII phosphorylates Ser^276^ of the autoinhibition domain, it inhibits the internal binding and promotes cargo interaction with PACS-1. PP2A, protein phosphatase 2A. **(D)** Each GGA is composed of four domains: VHS (pink), GAT (green), hinge (bar), and GAE (purple). Regulatory domain and sites of protein-protein interaction are shown. Autoinhibition sequence is shown (CKII phosphorylation site is in bold and the DXXLL motif is underlined in green). VHS, Vps27p, Hrs, Stam; GAT, GGA, and TOM; GAE, γ-adaptin ear. **(E)** Autoinhibition mechanism: when phosphorylated by CKII, the autoinhibition domain binds VHS preventing cargo interaction. When the autoinhibition is dephosphorylated, no internal binding occurs allowing cargo interaction with GGA. The single letter code for amino acids was used; CHC, clathrin heavy chain; CKII, casein kinase II.

Using the cell-free PC12 system, Arf1 was shown to enhance the formation of SGs and constitutive vesicles (42). Moreover, when Arf1 is bound to membranes isolated from PC12 cells, it recruits a set of different proteins, including the adaptor protein 1A (AP-1A) (54, 55). In non-endocrine secretory systems, such as rhoptries in *Toxoplasma gondii* (56), glue granules in *Drosophila* (57), and Weibel–Palade bodies in endothelial cells (58), AP-1A is required for the formation of SGs. Although it is not clear if AP-1A is required for SG formation in neuroendocrine cells, there is evidence that AP-1A is required for SG maturation (Figure 4A).

#### Membranes and SG formation: phosphatidylinositol-4-phosphate and cholesterol

The appropriate lipid composition is essential for normal TGN function and SG biogenesis. The TGN membrane is enriched in phosphatidylinositol-4-phosphate (PI4P). In mammalian cells, three enzymes that synthesize PI4P from phosphatidylinositol (PI) have been found at the Golgi, PI4KIIα, PI4KIIIα, and PI4KIIIβ; PI4KIIα (59) and PI4KIIIβ (60) have been implicated in export of material from the Golgi to the plasma membrane. PI4KIIα resides primarily in the TGN, endosomes, and SGs (61–64). Indeed, PI4KIIα was found on adrenal chromaffin granule membranes (65–67), cellugyrin-positive Glucose Transporter four vesicles (68), *Drosophila* salivary gland glue granules (69), synaptic vesicles (70), and immature SG membranes from PC12 cells (71). PI4KIIα is recruited to membranes upon palmitoylation of a central CCPCC motif (62). Deletion of the CCPCC motif or prevention of its palmitoylation resulted in loss of PI4KIIα perinuclear localization and loss of PI4KIIα activity (62). PI4KIIα palmitoylation is required for kinase activity and for localization to lipid rafts at the TGN but its targeting motif to Golgi membranes remains to be determined (63, 72). Lipid rafts are enriched in cholesterol and reduction of endogenous cholesterol levels resulted in loss of PI4P synthesis by PI4KIIα (63, 73). The membranes of neuroendocrine SGs contain high levels of cholesterol (74, 75). A decrease in the cholesterol level in AtT-20 cells inhibited the formation of constitutive secretory vesicles and SGs (76). Since PI4KIIα requires cholesterol for activity and membrane localization (63) and cholesterol is required for SG formation, this suggests that PI4KIIα is required for SG biogenesis.

## Maturation of SGs

Once immature SGs are formed, they are not responsive to secretagogues and must undergo a maturation process to gain this ability. SG maturation involves a decrease in lumenal pH, SG fusion, and membrane remodeling.

### pH decrease

The lumen of the TGN and the lumen of immature SGs are comparable in pH (pH 6.3), while mature SGs are more acidic (luminal pH 5.5). Secretogranin II processing in PC12 cells exogenously expressing prohormone convertase 2 occurs in immature SGs but not in the TGN (77). Although similar in lumenal pH, the TGN and immature SGs are two distinctly different compartments; the proteolytic processing of some prohormones and granins starts in immature SGs, not in the TGN. The decrease in pH during SG maturation is necessary for full processing of prohormones and granins because some hormone processing enzymes, such as PAM-1, exhibit maximal catalytic activity at pH 4.5–5 (78).

### SG fusion

Maturation of SGs can be characterized by a decrease or an increase in size. Analysis of immature and mature SGs in PC12 cells and mammotrophs revealed an increase in SG size during maturation (16, 79). An elegant biochemical assay revealed that the increase of SG size in PC12 cells was due to homotypic fusion of immature SGs (10). SG fusion involves syntaxin 6, a target (t)-SNARE [Soluble NSF (*N*-ethylmaleimide-Sensitive Factor) Associated Protein (SNAP) Receptor] protein, and synaptotagmin IV (80, 81). The SNARE complex is involved in membrane fusion and is composed of two t-SNAREs, located on the target membrane, and one vesicle (v)-SNARE on the vesicle membrane. Members of the synaptotagmin family are associated with the vesicular membrane and regulate membrane fusion with the SNARE complex. Syntaxin 6 localizes to the TGN, endosomes, and immature SGs (82, 83), while synaptotagmin IV is largely present at the Golgi and in immature SGs (84, 85). Blocking the fusion event decreases prohormone convertase 2 processing and activity, resulting in impaired SG maturation (80). Although this concept is attractive, there is no evidence of SG fusion in other types of neuroendocrine cells. SG fusion of a different type is clearly observed during compound exocytosis; mature SGs fuse together to promote rapid release of SG content (86).

### Membrane remodeling

The reduction of SG size during maturation may be explained by membrane remodeling. The presence of a clathrin coat on patches of immature SG membrane revealed egress of material in clathrin-coated vesicles, mediated by the AP, AP-1A (8, 9, 54). As a result, proteins like vesicle associated membrane protein 4 (VAMP4) (87, 88), furin (89), and both mannose 6-phosphate receptors (MPRs) (83, 90–92), each of which is known to have a canonical AP-1A binding site in its cytosolic domain, are found in immature SGs but not in mature SGs. Although the fate of the material retrieved from immature SGs is not entirely clear, there are lines of evidence suggesting that it can be recycled to the TGN or secreted in a process called constitutive-like secretion (Figure 1). The study of constitutive-like secretion is difficult to monitor since it refers only to the non-stimulated secretion of regulated SG proteins as they traverse immature SGs; without a means of assessing the time since synthesis, it is impossible to distinguish basal from constitutive-like secretion. Nevertheless, constitutive-like secretion has held many scientists’ attention due to its potential link to cancer and metabolic disease. Much of our knowledge of constitutive-like secretion comes from studying retrieval of material from immature SGs. The cytosolic machinery involved in this process contains the APs, AP-1A, and GGAs, and their partner phosphofurin acidic cluster sorting protein 1 (PACS-1). The discovery of an interaction between the cytosolic tail of membrane SG proteins retrieved during SG maturation with these cytosolic proteins has expanded our understanding of membrane remodeling during SG maturation. Each cytosolic component essential for maturation is presented below, followed by the description of SG membrane proteins trafficking involving AP-1A, GGAs, or PACS-1.

## Cytosolic Machinery

### Adaptor protein 1A

#### The adaptor protein family

The adaptor protein family includes five cytosolic heterotetrameric complexes: AP-1 (γ/β1/μ1/σ1), AP-2 (α/β2/μ2/σ2), AP-3 (δ/β3/μ3/σ3), AP-4 (ɛ/β4/μ4/σ4), and AP-5 (ζ/β5/μ5/σ5) (93–96). In mammals, several isoforms have been reported for subunits of AP-1, AP-2, and AP-3: AP-1 has two γ subunits (γ1 and γ2), two μ subunits (μ1A and μ1B), and three σ subunits (σ1A, σ1B, and σ1C); AP-2 has two α subunits (α1 and α2); AP-3 has two β subunits (β3A and β3B), two μ subunits (μ3A and μ3B); and two σ subunits (σ3A and σ3B) (93, 97–101). Every subunit is ubiquitously expressed except for μ1B, which is found exclusively in polarized epithelial cells, and β3B and μ3B, which are only found in neurons and neuroendocrine cells (99–101). Each adaptor protein carries transmembrane proteins on a defined intracellular route: AP-1 brings cargo between the TGN and endosomes and removes material from immature SGs; AP-2 is an important player in clathrin-mediated endocytosis; AP-3 carries cargo to lysosomes and lysosome-related organelles; AP-4 transports cargo from the TGN to the plasma membrane or endosomes; AP-5 is found on late endosomal membranes.

The cytosolic domains of cargo proteins present motifs which are recognized by adaptor protein complexes. For example, the Tyr sorting motif (YXXΦ, where X is any residue and ϕ is a hydrophobic residue) is recognized by the μ subunit of all adaptor protein complexes, with the possible exception of AP-5, which has not yet been studied (Figure 4A) (102–105). The di-leucine sorting motif [(D/E)XXXL(L/I)] interacts at the interface of two subunits: γ/σ1 in AP-1, α/σ2 in AP-2, and ɛ/σ3 in AP-3 (Figure 4A) (106, 107); AP-4 and AP-5 have not been shown to interact with the di-leucine motif. The γ/α/δ/ɛ/ζ and β1-5 subunits contain a large N-terminal trunk domain that associates with the other three subunits of the complex, followed by a hinge region and a C-terminal ear domain, which can interact with cytosolic proteins (Figure 4A) (108–111). The hinge region of the β subunits of AP-1, AP-2, and AP-3 is capable of binding the terminal domain of the clathrin heavy chain *in vitro*, while the β subunits of AP-4 and AP-5 lack such a motif (Figure 4A) (112–114). Although the β3 subunit of AP-3 can bind clathrin, purified clathrin-coated vesicles lack AP-3, suggesting that AP-3 works independently of clathrin (115). In addition, there is also evidence that the γ and α hinge domains of AP-1 and AP-2 interact with clathrin (Figure 4A) (116, 117).

#### AP-1A recruitment to membranes depends on Arf1 and PI4P

Adaptor protein 1A (γ/β1/μ1A/σ1) accumulates at the TGN, which is enriched in PI4P. *In vitro* binding assays revealed that purified AP-1A interacts preferentially with PI4P (Figure 4A) (59). Indeed, structural studies on the AP-1A core confirmed the presence of a PI4P binding site within the γ subunit (118). Reduction of PI4KIIα levels in a monkey kidney cell line decreased the TGN content of PI4P and disrupted the TGN localization of AP-1A (59). However, a recent study on development of the *Drosophila* salivary gland revealed no change in the localization of AP-1 in PI4KIIα null flies, suggesting that PI4P synthesis in flies can be carried out by another PI4K (69). Although the presence of PI4KIIα on the membranes of immature SGs suggests that PI4P can be formed there, there is no evidence that AP-1A recruitment to immature SGs requires PI4P in neuroendocrine cells.

Brefeldin A treatment of intact cells produces a diffuse cytoplasmic localization of AP-1A instead of the normal perinuclear, membrane-associated localization. However, pretreatment with GTPγS, a non-hydrolyzable analog of GTP, prevented the membrane dissociation of AP-1A upon addition of brefeldin A in permeabilized normal rat kidney cells (119). Indeed, AP-1A is recruited to membranes upon the binding of GTP-bound Arf1 to the trunk region of its β1 and γ subunits (Figure 4A) (55, 120–122). Studies on PC12 cells also show that AP-1A recruitment to immature SG membranes requires Arf1 (54, 55). Interestingly, AP-1A cannot be recruited to mature SG membranes in PC12 cells, in agreement with morphological studies indicating that AP-1A/clathrin coats are not found on mature SGs (55). A recent structural study of AP-1A in complex with GTP-bound Arf1 revealed that Arf1 can change the conformation of μ1A without the presence of PI4P or cargo; the addition of cargo promotes this conformational change (120). This shift in μ1A conformation is thought to promote the association of μ1A with cargo.

In addition to Arf1 and PI4P, AP-1A phosphorylation affects its localization; cytosolic AP-1A is phosphorylated on its β1 subunit, while membrane-associated AP-1A is phosphorylated on its μ1A subunit (123). Phosphorylation of the β1 subunit prevents clathrin binding, while phosphorylation of μ1A may promote conformational changes in the subunit that enable it to interact with cargo (118, 124). The phosphorylation status of AP-1A is thus essential for protein trafficking.

### Phosphofurin acidic cluster sorting protein 1 – an enhancer of AP-1A function

Adaptor protein 1A is not the only AP involved in the retrieval of material from immature SGs. Furin, a type I transmembrane endoprotease, cleaves peptides after the last Arg of an R-X-[K/R]-R motif; its discovery was a key advance contributing to the identification of the prohormone convertases that are essential to SG function. Furin is concentrated in the TGN and travels between the TGN, endosomes, and plasma membrane (125). In cells containing a regulated secretory pathway, furin was identified in immature SGs but not in mature SGs (89). The removal of furin from immature SGs involves AP-1A and clathrin-coated vesicles (89). Its cytoplasmic domain contains a Tyr-based sorting motif and an acidic cluster, which includes two Ser residues, both of which are substrates for CKII (Table 1) (126, 127). The Tyr motif found in the cytosolic domain of furin mediates TGN exit and plasma membrane internalization; the acidic cluster is important for retrieval of furin from endosomes and immature SGs (89, 127, 128). Mutation of the two CKII phosphorylation sites in furin to Ala (to prevent phosphorylation) resulted in the accumulation in mature SGs in a corticotrope tumor cell line (89, 129). Since AP-1A does not bind to acidic cluster, another interactor was sought. A search for proteins with high affinity for the phosphorylated acidic cluster through yeast two-hybrid screens led to the discovery of PACS-1 (127).

**Figure 5:**
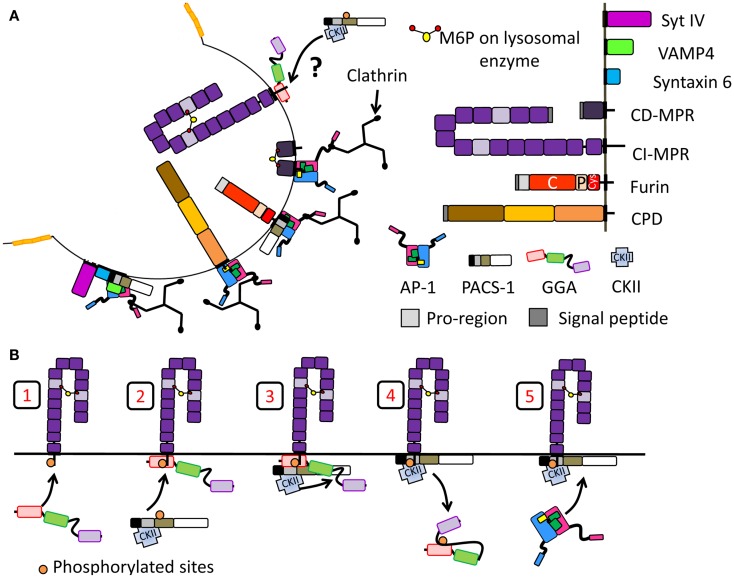
**Segregation of proteins at the immature SG during membrane remodeling**. Schematic of proteins introduced in Figure 2 are reused. **(A)** Proteins removed during SG maturation involving the recruitment of clathrin and clathrin adaptor protein. Proteins shown to interact with AP-1 and/or PACS-1 or GGAs are shown. In the case of CI-MPR, it is not clear whether GGAs help the recruitment of PACS-1 and AP-1 thus promoting its retrieval. **(B)** Model of adaptor protein recruitment for CI-MPR transport: GGA3 binds CI-MPR cytosolic tail (step 1). Then, PACS-1, presumably already interacting with CKII, is recruited to the GGA3/cargo complex (step 2). CKII phosphorylates the GGA3 hinge autoinhibitory region (step 3) resulting in GGA3 dissociation from membranes (step 4). PACS-1 then recruits AP-1 for the CI-MPR transport (step 5). Model adapted from (174).

Phosphofurin acidic cluster sorting protein 1 is a ubiquitously expressed cytosolic protein comprised of four domains: at its N-terminus is an atrophin-1-related region (ARR), with limited homology to atrophin-1; this region is followed by a furin binding region (FBR), the middle region (MR), and the C-terminal region (CTR) (Figure 4B) (127). The functions of the ARR and the CTR are unknown. The FBR of PACS-1 interacts with acidic clusters in cargo proteins, with APs (AP-1A, AP-3, and GGA), and with CKII (Figure 4B) (127, 129, 130). The MR contains an autoinhibition domain, which is composed of an acidic cluster with a CKII phosphorylation site (Figure 4C); when this region is not phosphorylated, the MR binds the FBR, resulting in inhibition of PACS-1 binding to its cargo (131). When the MR is phosphorylated by CKII, it reduces MR affinity for FBR, allowing the FBR to bind to acidic clusters in cargo proteins (130, 131). Furin has been identified in a heterotrimeric complex with AP-1A and PACS-1 (129). Expression of a PACS-1 mutant capable of binding the furin acidic cluster, but not AP-1A, does not alter the distribution of AP-1A, instead resulting in the accumulation of furin in mature SGs (129). This demonstrates that removal of furin from immature SGs is a cooperative process involving both AP-1A and PACS-1 (Figure 5A).

### GGAs – another family of adaptor proteins essential for SG maturation

Golgi-localizing, γ-adaptin ear homology domain, ARF-binding proteins (GGAs) were discovered by several groups interested in different aspects of the proteins. Boman et al. screened for Arf3 binding partners using a yeast two-hybrid system, while Hirst et al. used a bioinformatic approach to look for proteins with homology to the ear region of the γ subunit of AP-1 (109, 132). These studies, combined with the fact that the proteins discovered concentrated in the TGN area, explain the origin of the name GGA (109, 133). The GGA family contains three members in humans (GGA1, GGA2, and GGA3), two in *Saccharomyces cerevisiae* (Gga1p and Gga2p), and one in *Drosophila melanogaster* and *Caenorhabditis elegans* (109, 132, 134). Each member is ubiquitously expressed and acts as a monomeric clathrin adaptor (132, 135). From their N-terminus to their C-terminus, they are composed of a VHS [vacuolar protein sorting 27 (Vps27), hepatocyte-growth factor-receptor substrate (Hrs), signal-transducing adaptor molecule (Stam)] domain, a GAT [GGA and TOM (Target of Myb)] domain, a hinge, and a GAE (γ-adaptin ear) domain (134) (Figure 4D).

The VHS domain was identified based on its role in the trafficking of yeast Vps27p and for its homology to two other monomeric endosome-localized clathrin APs, Hrs, and Stam (109, 132, 136). A yeast two-hybrid screen revealed that the cytosolic domains of proteins containing either a tyrosine sorting motif (YXXΦ) or a di-leucine sorting motif [(D/E)XXXL(L/I)] were not recognized by any GGA proteins, but were specific to adaptor protein complexes (126, 137). The VHS domain of GGAs recognize a motif called acidic cluster-di-leucine motif (DXXLL), which is different from the adaptor protein di-leucine sorting motif [(D/E]XXXL(L/I)] (Figure 4D) (134, 137).

Target of Myb1 (TOM1), a protein involved in the trafficking of ubiquitinated proteins, contains a GAT domain similar to the GAT domain in GGAs. Indeed, both yeast Gga1p and mammalian GGAs bind monoubiquitin via their GAT domain (134, 138–141). This interaction is required for the sorting of some ubiquitinated cargo proteins in *S. cerevisiae*, such as Gap1 and Arn1 (134, 138, 142, 143). The GAT domain interacts with GTP-bound Arf1 and Arf3 and the C-terminus of GAT binds PI4P; as for AP-1A, both binding sites are required for proper membrane localization of GGA in cells (Figure 4D) (132, 133, 141). Indeed, brefeldin A treatment results in the dissociation of GGAs from TGN membranes and in their accumulation in the cytosol, as seen for AP-1A (109, 132, 133, 135). Interestingly, association of the GAT domain of GGA3 to Arf1 blocks GAP proteins from acting on GTP-bound Arf1, thus stabilizing it on TGN membranes (53).

The hinge region between the GAT and the GAE domains is predicted to lack secondary structure (134) and contains a clathrin binding motif which is necessary for the recruitment of clathrin *in vitro* and in cells (Figure 4D) (53, 109). While the GAE domain of GGA1 also interacts with clathrin heavy chain *in vitro*, the GAE domains of GGA2 and GGA3 are unable to do so (53). GGAs are important for the recruitment of clathrin to membranes; overexpression of GGAs increases the TGN localization of clathrin while overexpression of truncated GGA1 lacking the hinge and GAE domains reduces the TGN level of clathrin (53). Moreover, *in vitro* studies revealed that the GGA1-hinge region and the γ subunit of AP-1 interact (Figures 4A,D) (108, 144). In addition, the hinge region of both GGA1 and GGA3 contains a DXXL[L/M] motif, which can bind the VHS domain when the surrounding Ser residue has been phosphorylated by CKII (Figures 4D,E) (108, 145); this internal binding results in autoinhibition of the AP by loss of cargo binding (108, 145, 146). The autoinhibition is removed when the Ser residue is dephosphorylated by a protein phosphatase 2A-like enzyme (146). *In vitro* phosphorylation by CKII was not observed for GGA2, suggesting that GGA2 is controlled differently (108, 126). Although the autoinhibition concept is attractive, a recent structural report refutes the existence of such a motif within GGA1 and possibly within GGA3 as well (147). These conflicting results are currently unclear, but emphasize the fact that our knowledge of GGAs is incomplete.

The GAE domain has homology to the ear domain of γ-adaptin of AP-1 (109, 132, 133, 135, 148). It interacts with several cytosolic proteins including rabaptin-5, a Rab4/Rab5 effector involved in membrane fusion at the endosomes (109, 149, 150) and γ-synergin (148).

In neuroendocrine cells, reducing the levels of GGA3 or overexpressing a dominant negative form of GGA1 resulted in impaired egress of syntaxin 6, cation-independent MPR (CI-MPR), and VAMP4 from immature SGs and in decreased proteolytic processing of prohormone convertase 2 and secretogranin II (151). Thus GGAs, AP-1A, and PACS-1 are all key components in the maturation of SGs in neuroendocrine cells.

### Do GGAs and AP-1A work together?

Despite differences in their cargo recognition motifs, GGAs and AP-1A are both recruited to TGN membranes in an Arf1 and PI4P dependent manner, interact with clathrin and share common cargoes, such as CI-MPR (Table 1). Using transmission electron microscopy, vesicles budding from the TGN were seen to contain one AP or both GGA2 and AP-1A (108). A novel technique developed in mammalian cells to rapidly inactivate AP-1A or GGA2 revealed that AP-1A is the main AP required for trafficking between the TGN and endosomes; when AP-1A is inactivated, GGA2 is no longer present in clathrin-coated vesicles (152). In addition, GGA2 is thought to be required for proper localization of rabaptin-5, a group of lysosomal hydrolases, both types of MPRs and sortilin in clathrin-coated vesicles. Other substrates, such as Arf1, carboxypeptidase D (CPD), furin, the copper transporters (ATP7A and ATP7B), or the SNARE proteins require AP-1A for localization in clathrin-coated vesicles (152). In agreement with these data, mutation of the GGA binding site in the cytosolic tail of the CI-MPR decreases its amount in clathrin coats at the TGN, suggesting that GGAs help recruit specific cargo with APs and clathrin (108). However, a recent study in yeast using fluorescently tagged proteins suggested that the Gga proteins come into play first, and then recruit other proteins along with AP-1A after being released from the membrane (153). The yeast study also suggested that AP-1 and Gga proteins work mainly in separate vesicles. This divergence between yeast and mammals is unclear and may be linked to changes during evolution. Nevertheless, these studies do not provide information on the mechanism which governs the egress of material at the immature SG. In addition, yeast do not have a regulated secretory pathway, limiting their use in understanding SG maturation.

## Real Life Examples

Most of the integral membrane proteins found in post-TGN membranes contain multiple signaling motifs in their cytosolic tails. The study of protein retrieval from immature SGs was facilitated by our growing knowledge of APs and the motifs they recognize. While our understanding of the rules governing retention vs. retrieval of membrane proteins from immature SGs is not yet complete, detailed studies of the interactions of several immature SG cargo protein cytosolic tails with multiple APs reveals many of the key features (Figure 5A; Table 1).

### SNARE proteins

Similar observations to furin were made with the v-SNARE component, VAMP4 which contains an AP-1A binding motif (di-leucine sorting motif) and an acidic cluster surrounding a CKII phosphorylation site (Table 1) (87). VAMP4 accumulates at the TGN, on endosomes and on immature SG of PC12 and AtT-20 cell lines (87, 154). As for furin, overexpression of PACS-1 mutant unable to interact with AP-1A but capable of interacting with VAMP4 resulted in accumulation of VAMP4 in mature SG (87). Interestingly, VAMP4 accumulates in mature SGs only if both its AP-1A and its PACS-1 binding motifs are mutated; if either binding motif is remaining, VAMP4 is efficiently removed from immature SGs (87). This is different from furin which accumulated in mature SGs when the CKII sites were mutated. The AP-1A/PACS-1/clathrin sorting system seems to work differently with VAMP4 and furin.

In addition, VAMP4 interacts with syntaxin 6, which binds synaptotagmin IV (80, 154). All three proteins are retrieved from immature SGs (83, 84, 154). Although *in vitro* studies failed to show binding between syntaxin 6 and AP-1A (87), observation of immature SGs using electron microscopy showed that syntaxin 6 is retrieved in AP-1A and clathrin containing vesicles (83). It remains unclear whether synaptotagmin IV interacts with AP-1A, but since the removal of VAMP4 and syntaxin 6 has been linked with AP-1A, it seems likely that synaptotagmin IV removal is also dependent on the AP-1A/PACS-1/clathrin sorting system due to its indirect binding with VAMP4. Interestingly synaptotagmin IV is known to inhibit calcium triggered exocytosis (84), suggesting that its removal is necessary to make mature SG responsive to secretagogues. Since synaptotagmin IV plays a critical role in SG fusion which is an essential step in SG maturation, these studies would suggest that SG fusion occurs before membrane remodeling.

### The mannose 6-phosphate receptors

Mannose 6-phosphate receptors transport soluble lysosomal hydrolases from the TGN to endosomes (137, 155). MPRs are found in immature SGs and are removed during maturation in clathrin-coated vesicles that contain AP-1A (83). Two MPRs have been described: MPR300, the CI-MPR, and MPR46, the cation-dependent MPR (CD-MPR). Much has been learned by studying the multiple trafficking signals in both MPRs. Both MPRs contain Tyr sorting motifs (YXXΦ) and an acidic cluster-di-leucine motif (DXXLL) next to a CKII phosphorylation site (Table 1). Early studies revealed that the Tyr motif of CI-MPR is recognized by AP-2 but not by AP-1A (156). CD-MPR contains two tyrosine motifs: the first tyrosine motif is located next to the transmembrane domain, and may not be a signaling motif because of the hindrance caused by the membrane (Table 1, Y^211^QRL). The second tyrosine motif is found away from the membrane (Table 1, Y^256^RGV) and is implicated in the protein internalization (157).

The DXXLL motif is recognized by the VHS domain of GGAs (137, 150). Interestingly, the DXXLL motif found on the cytosolic tail of the CI-MPR binds the VHS domain of all GGAs while the DXXLL motif of the CD-MPR interacts with the VHS domain of GGA1, interacts weakly with GGA3 and does not interact with GGA2 (137, 150). Mutational analyses revealed that the residues surrounding the motif are important for establishing GGA specificity (137). Down-regulation of GGA3 or inhibition of GGA1 results in the accumulation of CI-MPR in mature SGs, suggesting that retrieval of CI-MPR from immature SGs requires GGAs (151).

Independent *in vitro* studies focusing on either CD-MPR or CI-MPR showed that removal of the di-leucine motif from either receptor had little effect on its ability to recruit AP-1A. In CI-MPR, elimination of the CKII phosphorylation site abolished recruitment of AP-1A (90, 127). Indeed, binding of AP-1A to CI-MPR requires prior phosphorylation of its cytosolic domain by CKII (91, 158). These features suggested that PACS-1 might be involved in the trafficking of CI-MPR. In non-neuroendocrine cells, reducing the cellular level of PACS-1 or overexpressing a PACS-1 mutant unable to interact with acidic clusters resulted in accumulation of CI-MPR in endocytic compartments, suggesting that PACS-1 recruitment is necessary for MPR retrieval from the endosomes to the TGN (127, 129). AP-1A binding to CD-MPR requires the acidic cluster; experiments aimed at understanding the role of phosphorylation at the Ser CKII site in CD-MPR in its binding of AP-1A have yielded conflicting results (Table 1, Ser^268^) (91, 159). It is not yet known whether MPRs are retrieved from immature SGs in a PACS-1 dependent manner.

GGA3 and PACS-1 interact with the CI-MPR at sites that are overlapping, but not identical (Table 1) (130). The FBR region of PACS-1 binds CKII, enhancing its kinase activity (130). This results in phosphorylation of the MR of PACS-1, releasing the FBR for cargo interaction (Figure 4C). In addition, CKII phosphorylates GGA3, decreasing its affinity for cargo and thus making space for PACS-1 to bind (Figures 4E and 5B) (130). Although this study did not focus on immature SGs, it provides a mechanism of why GGA and AP-1A/PACS-1 are necessary for SG maturation.

### Carboxypeptidase D does not contain an AP-1A binding motif

The exopeptidase CPD, a type I transmembrane protein, accumulates in the TGN and cycles between the TGN and plasma membrane via endosomes (160). In AtT-20 cells, CPD is found in immature, but not in mature SGs suggesting that it is removed during SG maturation (161). The isolated luminal domain of CPD primarily enters the constitutive secretory pathway, suggesting that signals in its cytosolic tail play an essential role in CPD entry into immature SGs and in the TGN retention of CPD (161). The CPD cytosolic tail contains an acidic cluster with two CKII phosphorylation sites (TDT), but lacks classical AP and GGA binding motifs (Table 1). *In vitro* binding assays revealed that the CPD cytosolic tail binds to AP-1A and AP-2 only when its two CKII phosphorylation sites are phosphorylated or have been mutated to phosphomimetic residues (EDE) (Table 2) (162). Deletion of the C-terminus of the CPD tail, which contains the CKII phosphorylation sites does not abolish AP-1A or AP-2 binding (Table 2) (162), suggesting a role for additional CPD/AP-1A/2 interaction motifs. A tyrosine-like motif (Table 2, F^1332^HRL) located upstream of the acidic cluster is thought to be important for TGN export and endocytosis and is necessary for AP-1A and AP-2 binding (162). In addition, the C-terminus of CPD may bind another cytosolic protein which would prevent APs to interact. This unidentified protein probably loses its affinity for the C-terminus of CPD when the CKII sites are phosphorylated or removed, allowing APs to interact. Phosphorylation of CPD by CKII promotes AP-1A binding, suggesting that PACS-1 plays a role in its trafficking. The trafficking of CPD at immature SGs probably results from the coordinated work of multiple cytosolic proteins including AP-1A and PACS-1.

**Table 2:** **Summary of mutational analysis of CPD tail**.

Protein	Sequence			AP-1A binding
CPD-tail full, not phosphorylated	*TMD*	C^1319^ICSIKSNRHKDGFHRL^1335^ […] S^1356^LLSHEFQDE**T**D**T**EEETLYSSKH^1378^	*COOH*	−
CPD-tail full, phosphomimetic	*TMD*	C^1319^ICSIKSNRHKDGFHRL^1335^ […] S^1356^LLSHEFQDE**E**D**E**EEETLYSSKH^1378^	*COOH*	**++**
CPD-tail missing the last 18 residues	*TMD*	C^1319^ICSIKSNRHKDGFHRL^1335^ […] S^1356^LLSH^1360^	*COOH*	**+**

Results adapted from (162). TMD, transmembrane domain; COOH, C-terminus of the protein. CKII phosphorylation site is in bold, the acidic cluster in red and the AP-1A-like site in blue. Gray marks TMD and COOH

### VMAT2 suggests the existence of additional adaptor proteins

The monoamine transporter, VMAT2, accumulates in mature SGs in neuroendocrine cells. Its cytosolic domain contains an acidic cluster with two CKII sites at the C-terminus; upon CKII phosphorylation, this site interacts with PACS-1 (163). As for furin, expression of VMAT2 in which the CKII sites have been replaced with phosphomimetic mutations promotes its retrieval from immature SGs. Mutation of these same two residues into Ala to prevent phosphorylation promotes VMAT2 accumulation in mature SGs (163); interestingly, expression of VMAT2 lacking the acidic cluster/CKII domain resulted in its egress from immature SGs (163). Unlike furin, which accumulates in the TGN at steady state, VMAT2 concentrates in mature SG raising the question of VMAT2 retention mechanism. These findings suggest that additional cytosolic proteins are involved in the sorting of SG proteins at the immature SG and may explain the retention of other membrane proteins in SGs during maturation. Indeed the amidating enzyme, PAM, remains in SGs during maturation, and presents an acidic cluster with two CKII phosphorylation sites (39). Although direct binding between PAM cytosolic domain and PACS-1 has not been shown, it is possible that PAM interacts with the same cytosolic proteins which bind VMAT2 for its retention in SG.

### Phogrin

Phogrin is a transmembrane protein that spans the membrane of immature and mature SGs. The cytosolic domain of phogrin contains a protein-tyrosine phosphatase (PTP) domain preceded by a 105 residue-long domain and followed by 10 residues at its C-terminus (Figure 2B; Table 1). The PTP domain of phogrin is an active phosphatidylinositol (PI) phosphatase; its enzymatic activity is thought to play an essential role in its ability to regulate peptide hormone secretion (164). Both domains surrounding the PTP domain are involved in the correct sorting of phogrin in the regulated secretory pathway (36, 37). The 105 residue stretch close to the transmembrane domain contains a tyrosine-extended motif (Table 1, Y^655^QEL) and the 10 residues at the C-terminus present a di-leucine-like motif (Table 1, E^993^EVNAIL). Both motifs are required for entry of phogrin into SGs and internalization at the plasma membrane (36, 37). Interestingly, both motifs are involved in the binding of phogrin to AP-1A and AP-2. Mutation of one of these motifs decreases the binding strength of APs for phogrin but does not abolish the interaction (36, 37). Although phogrin binds AP-1A via unconventional motifs, it still remains to be determined what prevents this transmembrane protein from being removed during SG maturation.

### Sortilin and pro-BDNF

Sortilin, a type I transmembrane protein, binds the pro-region of brain-derived neurotrophic factor (pro-BDNF) and transports it to SGs for processing (165). As for CPD, expression of the luminal domain of sortilin results in its entry into the constitutive secretory pathway, suggesting that entry into SGs is mediated by the cytosolic tail (166). A recent study revealed that interaction of sortilin with Huntingtin associated protein 1 (HAP-1) and pro-BDNF must occur for pro-BDNF entry into SGs and subsequent proteolytic processing (167). In the absence of HAP-1, sortilin, and pro-BDNF are targeted to lysosomes. Interestingly, the cytosolic tail of sortilin contains both a tyrosine motif and an acidic cluster-di-leucine motif, classical AP, and GGA binding sites, respectively (Table 1). Both motifs are implicated in sortilin internalization (168). As expected, the VHS domain of GGA2 interacts with the acidic cluster-di-leucine motif of sortilin (168). However, the tyrosine motif is recognized by the retromer complex, not by AP-1A (169). The AP-1A binding motif on sortilin remains to be elucidated. Both GGA2 and AP-1A were shown to be important for the TGN-to-endosome transport of sortilin. It is possible that HAP-1 interacts with sortilin to prevent GGA and AP-1A binding and mediates the entry of sortilin and pro-BDNF into SGs. Although sortilin contains AP binding motifs, it is not clear if sortilin is retrieved from immature SGs. One could assume that once in immature SGs, sortilin can release pro-BDNF and be retrieved during SG maturation.

## Conclusion and Future Directions

Little is known about the cytosolic machinery involved in sorting membrane proteins at the TGN for their entry into immature SGs. A recent study in *Drosophila* suggested that AP-3, the AP transporting proteins to lysosomes, was involved in sorting proteins into the regulated secretory pathway at the TGN level (170). Unfortunately, it is not known whether this is also true in the mammalian neuroendocrine system. In addition, studies on the trafficking of individual proteins raises many questions regarding the cytosolic machinery necessary for their retrieval or retention during SG maturation. It remains to be explained how a membrane protein which contains an AP (AP-1A, PACS-1, or GGAs) binding motif can remain in SGs during maturation. One possibility is that other cytosolic proteins bind the tails of membrane proteins, preventing their interaction with APs. Recent proteomic studies on purified SGs are an excellent source of information and may help identify additional proteins involved in the egress of membrane proteins (171, 172). Another possibility is a binding site within the cytosolic tail of membrane proteins which could prevent AP interaction; a post-translational modification, such as phosphorylation, can abolish this internal interaction while promoting the interaction with APs. Lastly, the membrane of the immature SG may play an important role in concentrating membrane proteins to be removed during maturation. Katsumata et al. addressed this question using the rat parotid gland as a source of SGs. The immature SG membrane revealed spatial segregation of membrane proteins: VAMP2 and Rab3D were in GM1a rich microdomains, while syntaxin 6 and VAMP4 were not. Since VAMP2 and Rab3D remain in mature SGs while syntaxin 6 and VAMP4 are removed from immature SGs, membrane rearrangement could explain how the right proteins are sorted for retrieval of material at the immature SG (173). The trafficking of individual proteins found in immature SGs can differ based on the cell type or animal model. It is evident that professional secretory cells share a common mechanism, and that the differences they present are probably required for their physiological function.

## Conflict of Interest Statement

The authors declare that the research was conducted in the absence of any commercial or financial relationships that could be construed as a potential conflict of interest.
